# Fahr’s Disease: A Differential to Be Considered for Various Neuropsychiatric Presentations

**DOI:** 10.7759/cureus.2304

**Published:** 2018-03-11

**Authors:** Seyedmohammad Pourshahid, Mohammad Nour Salloum, Mohanad Elfishawi, Mohamed Barakat, Mohammed Basith

**Affiliations:** 1 Internal Medicine, Icahn School of Medicine at Mount Sinai, Queens Hospital Center

**Keywords:** fahr disease, basal ganglia calcification, familial basal ganglia calcification

## Abstract

Fahr’s disease, also known as familial idiopathic basal ganglia calcification, is a neurodegenerative disorder affecting cerebral microvessels, mainly the basal ganglia, and presenting with diverse neuropsychiatric manifestations. It is considered to be mainly hereditary, with autosomal dominant inheritance. In light of its various presentations and incomplete penetrance, Fahr’s disease is known to be underestimated and underdiagnosed. Here, an early-onset case of Fahr’s disease is presented mainly with pure psychiatric symptoms. Given the diversity of the presenting symptoms, and variations in the age of onset, further investigation of organic etiologies in patients presenting with neuropsychiatric symptoms, family members of patients with Fahr’s disease, and patients with unexplained cerebral calcification is recommended.

## Introduction

Fahr’s disease is considered to be one of the rare neurological diseases that present with a wide array of presentations, including movement disorders and psychiatric manifestations [1].The disease was previously described by the German neurologist Fahr in the early 1930s [2]. Since then, many scientists have been trying to develop a better understanding of this rare disease [3]. There is strong evidence to suggest that the disease is largely hereditary, with a possible autosomal dominant mode of inheritance. The age of onset is reported to be around the fourth decade of life. Several endocrine abnormalities were found to be associated with Fahr’s disease, especially with regard to calcium metabolism. The unique radiological finding of basal ganglia calcifications might be related to the dysfunctional calcium regulation [4].

## Case presentation

A 20-year-old female with past medical history of hypocalcemia was admitted to the hospital for abnormal behavior. She was found to be agitated and speaking incoherently by the police. Further history from family members revealed that the patient had been having paranoid delusions for the past two weeks. There was no history of psychiatric disorders, paranoid behaviour, or recent alcohol, tobacco or illicit substance use. The patient was not taking any medication or herbal preparations. Review of systems was unremarkable. There was no family history of psychiatric or neurological diseases from the mother’s side. Paternal family history was unavailable as the patient was estranged from her father.

Her vital signs on admission were within normal limits. On physical examination, the patient was uncooperative, agitated, and had slurred speech. Neurological exam revealed brisk patellar reflexes and an unsteady gait, but was otherwise normal. Laboratory investigations revealed haemoglobin (Hb) of 9.5 gm/dL, with a serum iron level of 44 µg/dL, serum ferritin 6.9 ng/mL, and total iron binding capacity (TIBC) greater than 450 µg/dL. Basic metabolic panel revealed serum ionized calcium of 3.7 mg/dL, potassium level of 4.4 mmol/L, and magnesium of 1.6 mg/dL. Evaluation of her hypocalcemia revealed PTH of 11.3 pg/mL, vitamin D 25 hydroxy of 11.8 ng/mL, TSH of 0.65 µIU/mL, and free T4 of 1.06 ng/dL. Liver function tests were within normal limits. Urine toxicology screen was negative for substance abuse. Ethanol, salicylates, acetaminophen, and tetrahydrocannabinol (THC) tests were all negative.

**Figure 1:**
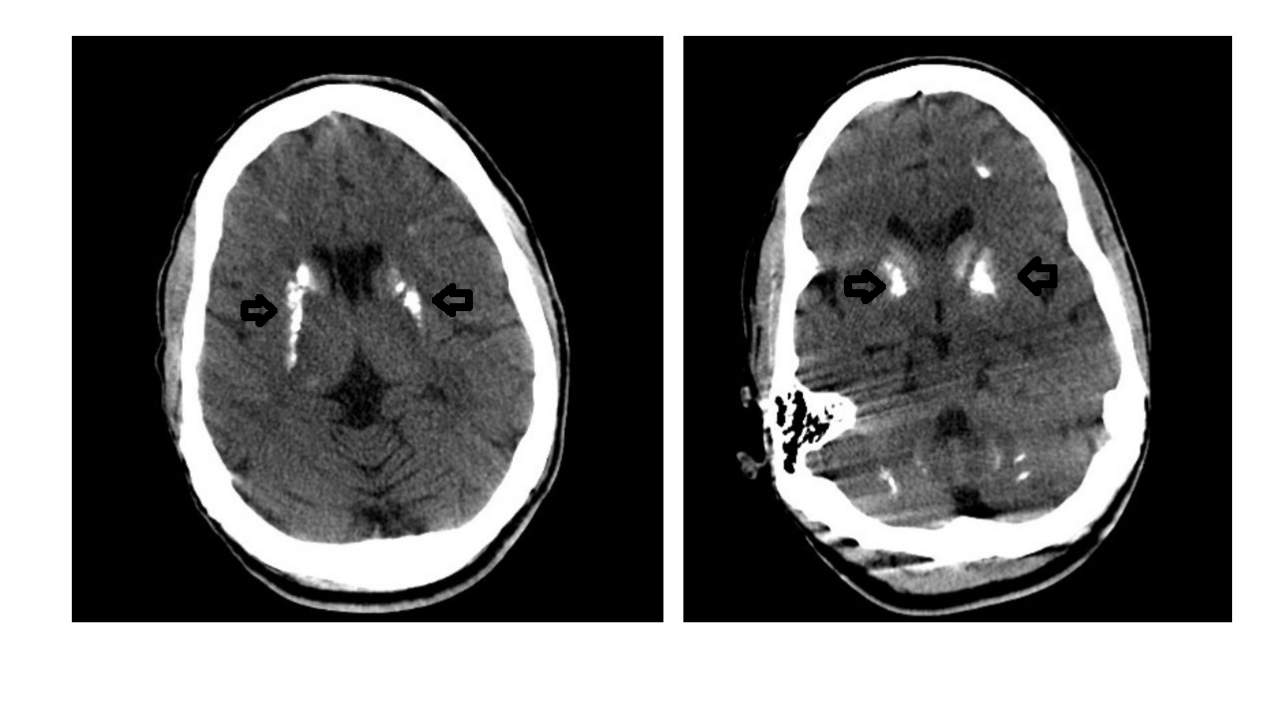
CT head Bilateral basal ganglia calcification as shown by the arrows.

Computed tomography (CT) of head was performed, which showed prominent basal ganglia calcifications, with additional scattered calcifications in the periventricular and subcortical white matter (Figure 1). Electro-encephalogram (EEG) did not reveal any abnormalities. These findings confirmed the diagnosis of Fahr’s disease.

The patient was treated with calcium and vitamin D supplementation. After normalization of her calcium level, her paranoid delusions and psychosis subsided. Follow up in clinic revealed that there was no recurrence in her symptoms.

## Discussion

Fahr’s disease, also known as familial idiopathic basal ganglia calcification, is a neurodegenerative disorder affecting cerebral microvessels, mainly the basal ganglia, and presenting with diverse neuropsychiatric manifestations. When the basal ganglia calcifications are secondary to a known cause, the disease is referred to as Fahr’s syndrome [1]. Beside the genetic etiology, metabolic derangements, infections, and other conditions are associated with the syndrome [2]. Familial cases are predominantly inherited in an autosomal dominant fashion. Estimating prevalence is challenging due to the vast diversity of presenting symptoms and incomplete penetrance of the disease. However, with the most conservative estimations, the minimal prevalence of variants of known genes is 4.5 p. 10,000 (95%CI [3.4–5.5] p. 10,000). Population genomic analysis reveals that this is not a very rare disease, and it has been underestimated and underdiagnosed so far [3].

The disease onset is usually in the fourth to fifth decade, with various neurological and psychiatric symptoms. Our patient presented at the age of 20, which is an unusually young age of presentation compared to patients with Fahr’s disease. Also, isolated psychiatric symptoms without neurological manifestations are rarely seen in patients with Fahr’s disease. Bilateral basal ganglia calcifications are usually present. Symptoms include progressive neuropsychiatric findings including dementia, delirium, confusion, hallucinations, psychosis, mood disorders, panic attacks, irritability, and aggression. Somatic symptoms such as Parkinson-like movement disorder, seizure, headache, stroke, syncope, and tremor might also be present [2,4-5].

Metastatic disposition of calcium, local disruption of the blood-brain barrier, or disorders of neuronal calcium metabolism are some of the possible causes for brain calcifications [5]. The association of calcium dysregulation, signaling and disturbed homeostasis and psychiatric disorders like schizophrenia and bipolar disorder was hypothesized [6]. Also, several disorders involving the basal ganglia like Parkinson’s disease and Wilson’s disease present with neuropsychiatric symptoms, in addition to movement disorders [7].

Fahr’s disease presenting with such common neuropsychiatric symptoms like mood disorders, cognitive disorders, hallucinations, and delusions is a differential diagnosis that needs to be ruled out [8]. On the other hand, the differential diagnosis for basal ganglia calcification is broad, including neoplasms like oligodendrogliomas, astrocytomas, medulloblastomas, and metastatic tumors; certain infections like TORCH (toxoplasmosis, other [syphilis, varicella-zoster, parvovirus B19], rubella, cytomegalovirus, and herpes) infections, tuberculosis and parasitic infections; vascular etiologies like angiomatous malformations, arteriovenous malformations, and chronic vasculitis; congenital syndromes like Sturge-Weber syndrome, tuberous sclerosis, and neurofibromatosis; metabolic causes like diabetes mellitus, hyperparathyroidism, and pseudohyperparathyrodisim. Brain calcifications, although interpreted as of no clinical significance, and seen as an incidental finding by some physicians, has a broad differential diagnosis and particularly needs to be carefully evaluated for underlying etiologies, especially in patients below the age of 30 years [9].

Currently, symptomatic treatment is the only option available for Fahr’s disease patients, but treatment of associated conditions like hypoparathyroidism has been shown to improve neuropsychiatric symptoms [10].

## Conclusions

In conclusion, Fahr’s disease is a neurodegenerative disorder presenting with a wide array of neuropsychiatric symptoms and is underestimated and underdiagnosed. Further investigation of organic etiologies in patients presenting with neuropsychiatric symptoms, family members of patients with Fahr’s disease, and patients with evidence of cerebral calcification in imaging is recommended.
